# Correction: Evolving Trends in the Management of Acute Appendicitis During COVID-19 Waves: The ACIE Appy II Study

**DOI:** 10.1007/s00268-022-06808-2

**Published:** 2022-10-22

**Authors:** Francesco Pata, Marcello Di Martino, Mauro Podda, Salomone Di Saverio, Benedetto Ielpo, Gianluca Pellino, the ACIE Appy Study Collaborative, Abba Julio, Abdullah Alshamrani, Abdullah Alturkistani, Abdulrahman Alghamdi, Abdulrahman Almalki, Adam Orengia, Adnan Kuvvetli, Adolfo Pisanu, Adrian Smith, Adriana Michelle Treviño Figueroa, Aeris Jane Nacion, Ahmad Alhazmi, Ahmad Bouhuwaish, Ahmad Khalid, Ahmed Alsufyani, Ainhoa Valle Rubio, Akshay Bavikatte, Akshay Kumar, Al-Radjid Jamiri, Alberto de San Ildefonso Pereira, Alberto Porcu, Alberto Sartori, Aldo Rocca, Aleksandar Sretenovic, Alessandro Anselmo, Alessandro De Luca, Alexandros Charalabopoulos, Alexios Tzivanakis, Alfonso Bandin, Alfonso Nájar, Alice Frontali, Alsulaimani Faisal, Amaia Martínez Roldan, Amal Hamid, Ana André, Ana Minaya-Bravo, Andre Das, Andrea Bondurri, Andrea Costanzi, Andrea Lucchi, Andrei Mihailescu, Andrea Police, Andres Mendoza Zuchini, Angela Romano, Angelo Iossa, Antonella Chessa, Antonella Tromba, Antonio Castaldi, Antonio Brillantino, Antonio Ferronetti, Antonio Giuliani, Antonio Ramos-De la Medina, Antonio Tarasconi, Arcangelo Picciariello, Argyrios Ioannidis, Ari Leppäniemi, Arshad Rashid, Ashrarur Rahman Mitul, Asif Mehraj, Asim laharwal, Atif Iqbal, Athanasios Liarakos, Athanasios Marinis, Beatriz de Andrés-Asenjo, Belén Matías-García, Belinda De Simone, Ben Creavin, Ben Stubbs, Brian Goh, Branislav Jovanovic, Bruno Sensi, Carlo Gazia, Carlos Cerdán, Carlos Javier Gómez Díaz, Carlos Petrola Chacón, Carlos Yánez, Carmelo Lo Faro, Caroline Reinke, Casandra Dominguez, Charudutt Paranjape, Charlotte Thomas, Chia Chi Fung, Chiara De Lucia, Chiu Hiu Fung Jennifer, Christian Ovalle-Chao, Claudio Guerci, Cleo Kenington, Corina Gica, Cristina Folliero, Cristopher Varela, Daniel Popowich, Daniele Delogu, Daniele Zigiotto, Danilo Vinci, Dario D’Antonio, David Alessio Merlini, David Merlini, David Moro-Valdezate, Deborah Keller, Diana Cristiana Nicolaescu, Diego Sasia, Edgar Rodas, Dimitrios Linardoutsos, Domenico Russello, Pedro Alfonso Nájar-Castañeda, Habil Gregor Stavrou, Edoardo Rosso, Edoardo Saladino, Edoardo Ricciardi, Eduardo Smith-Singares, Efstratia Baili, Eleftheria Douka, Eleonora Guaitoli, Elisa Francone, Elisa Maria Vaterlini, Elisa Sefora Pierobon, Emilio Morales, Emilio Peña Ros, Enrico Benzoni, Enrico Erdas, Enrico Pinotti, Enrique Colás-Ruiz, Ernesto Laterza, Esteban Foianini, Eugenia Cardamone, Eugenio Licardie, Fabio Marino, Fahad Alsofyani, Fahad Qahtani, Farhan Khan, Fatlum Maraska, Fatmir Saliu, Fausto Madrid, Fausto Rosa, Federico Luvisetto, Felipe Alconchel, Felipe Pareja-Ciuro, Fernanda Neves, Ferdinando Agresta, Fernando Cordera, Fernando Pardo, Fernando Mendoza-Moreno, Fernando Munoz-Flores, Francesca Maria Silvestri, Francesca Paola Tropeano, Francesca Pecchini, Francesca Serio, Francesco Colombo, Francesco Di Marzo, Francesco Ferrara, Francesco Lancellotti, Francesco Litta, Francesco Martini, Francesco Roscio, Francisco Blanco-Antona, Francisco Javier Quezada Barcenas, Francisco Schlottmann, Gabriel Herrera-Almario, Gabrielle van Ramshorst, Gaetano Gallo, Gaetano Luglio, Georgios Kampouroglou, Georgios Papadopoulos, Gerardo Arredondo, Giacomo Calini, Giampaolo Formisano, Giampaolo Galiffa, Gian Marco Palini, Gianluca Colucci, Gianluca Pagano, Gianluca Vanni, Gianmaria Casoni Pattacini, Gianpiero Gravante, Giorgio Lisi, Giovanni Bellanova, Giovanni De Nobili, Giovanni Sammy Necchi, Giovanni Sinibaldi, Giulia Bacchiocchi, Giulia Bagaglini, Giulia Maggi, Giuliano Izzo, Giulio Argenio, Giuseppe Brisinda, Giuseppe Esposito, Giuseppe Frazzetta, Giuseppe Massimiliano De Luca, Giuseppe Nigri, Giuseppe Sica, Gonzalo Martin-Martin, Gustavo Armand Ugon, Gustavo Martinez-Mier, Gustavo Miguel Machain Vega, Gustavo Nari, Herald Nikaj, Ignacio Neri, Igor Alberdi San Roman, Iliya Fidoshev, Iñaki Martínez, Ionut Negoi, Irene Ortega, Irune Vicente Rodríguez, Isabel Cornejo, Ismael Mora-Guzmán, Issam al-Najami, Ivan Romic, Izaskun Balciscueta, James Olivier, Jan Lammel-Lindemann, Jana Dziakova, Javier Salinas, Jelena Pejanovic Jovanovic, Jeryl Anne Silvia Reyes, Joanne Salas, Jose Antonio Diaz-Elizondo, Jose Gustavo Parreira, Juan Bellido, Juan Salamea, Juan Carlos Martín del Olmo, Juliana María Ordoñez, Sofi Junaid, Justin Davies, Kapil Sahnan, Kebebe Bekele, Kelvin Voon, Leandro Siragusa, Lorenzo Petagna, Luca Ferrario, Luca Giordano, Luca Nespoli, Luca Pio, Lucia Moletta, Luciano Curella, Lucio Taglietti, Luigi Bonavina, Luigi Conti, Luis Eduardo Pérez-Sánchez, Luis Felipe Cabrera Vargas, Luis Sánchez-Guillén, Luis Tallon-Aguilar, Mansoor Khan, Marcello Giuseppe Spampinato, Marcelo Viola, Marcelo Viola Malet, Marco Angrisani, Marco Calussi, Marco Catarci, Marco Giordano, Marco Materazzo, Marco Milone, Marco Pellicciaro, Marco Vito Marino, María Daniela Moreno Villamizar, Maria Giulia Lolli, Maria Irene Bellini, Maria Lemma, Maria Michela Chiarello, Mario Montes-Manrique, Mario Rodriguez-Lopez, Mario Serradilla-Martín, Mark Peter, Marta Paniagua-García-Señoráns, Martin Rutegård, Martin Salö, Massimiliano Silveri, Massimiliano Veroux, Matteo Nardi, Matteo Rottoli, Matti Tolonen, Mauricio Pedraza Ciro, Mauricio Zuluaga, Maurizio Iacobone, Mauro Montuori, Mazin Ali, Melody García Domínguez, Menna Maria Paola, Micaela Piccoli, Michela Campanelli, Michele De Rosa, Michele Manigrasso, Michele Maruccia, Michele Torre, Michele Zuolo, Miguel Pera, Mihiri Weerasekera, Mikel Prieto, Min Myat Thway, Mohamed Shaat, Mohammad Azfar, Mostafa Shalaby, Muhammad Asif Raza, Muhammad Umar Younis, Muhammed Elhadi, Mujahid Ali, Musab AlThomali, Nadiah Al Amri, Nagendra Dudi-Venkata, Nahar Alselaim, Neil Smart, Nelson Trelles, Nicolò Falco, Niccolo Petrucciani, Nicola Antonacci, Nicola Cillara, Nicolae Gica, Nicolò Pecorelli, Nicolò Tamini, Nikolaos Machairas, Nura Feituri, Nuria Ortega Torrecilla, Octavio Avila Mercado, Ohood AlAamer, Oktay Irkorucu, Omar Alsherif, Oreste Claudio Buonomo, Orestes Valles-Guerra, Orestis Ioannidis, Oscar Isaac Hernández Palmas, Oscar Sanz Guadarrama, Osman Bozbiyik, Pablo Rodrigues, Pamela Milito, Paolo Panaccio, Panagiotis Dorovinis, Paola Prieto, Paolo Baroffio, Patrizia Marsanic, Pawel Ajawin, Peng Soon Koh, Pietro Anoldo, Piotr Major, Qasem Alharthi, Rashid Lui, Riccardo Caruso, Richard Brady, Rishi Rattan, Rishi Singhal, Roberta Angelico, Roberta Maria Isernia, Roberta Tutino, Roberto Peltrini, Rodrigo Tejos, Roosevelt Fajardo, Rossella Elia, Salvador Morales-Conde, Sami Benli, Sara Fuentes, Sara Gortázar de las Casas, Sara Ortiz de Guzmán Aragón, Sara Vertaldi, Selmy Awad, Sergio Gentilli, Sergio Alberto Weckmann Lujan, Serkan Tayar, Shabab Althobaiti, Silvia Di Giovanni, Soliman Ghedan, Sonia Pérez-Bertólez, Sonja Chiappetta, Spiros Delis, Stefano Scaringi, Süleyman Çetinkünar, Stylianos Kykalos, Syed Muhammad Ali, Sylvia Krivan, Tak Lit Derek Fung, Tarik Delko, Tatiana Nicolás López, Tercio De Campos, Teresa Calderón Duque, Teresa Perra, Theodore Liakakos, Theodoros Daskalakis, Tijmen Koëter, Tiku Zalla, Tomás Elosua González, Tommaso Campagnaro, Toure Alpha Oumar, Ugo Grossi, Valentina Sosa, Valentina Testa, Valentina Tomajer, Valeria Andriola, Valeria Tonini, Valerio Celentano, Valerio Voglino, Venkateswara Rao Katta, Víctor Hugo García Orozco, Victor Turrado-Rodriguez, Victor Visag-Castillo, Victoria Graham, Viktor Rachkov, Vincenzo Papagni, Vincenzo Vigorita, Virginia Jiménez Carneros, Vittoria Bellato, Wolf Bechstein, Yuksel Altinel, Zutoia Balciscueta

**Affiliations:** 1 General Surgery Unit, UOC di Chirurgia, Nicola Giannettasio Hospital, Via Ippocrate, 87064 Corigliano-Rossano, CS Italy; 2 La Sapienza University, Rome, Italy; 3 Division of Hepatobiliary and Liver Transplantation Surgery, A.O.R.N. Cardarelli, Naples, Italy; 4 Department of Surgical Science, University of Cagliari, Cagliari, Italy; 5 Department of Surgery, Madonna del Soccorso General Hospital, San Benedetto del Tronto, Italy; 6 Hepatobiliary Division, Hospital del Mar, Universitat Pompeu Fabra, Barcelona, Spain; 7 Department of Advanced Medical and Surgical Sciences, Universitá degli Studi della Campania “Luigi Vanvitelli”, Policlinico CS, Piazza Miraglia 2, 80138 Naples, Italy; 8 Colorectal Surgery, Vall d’Hebron University Hospital, Barcelona, Spain

## Correction to: World J Surg https://doi.org/10.1007/s00268-022-06649-z

In the original online version of this article Oreste Claudio Buonomo's family name was misspelled.

The original article was corrected.

